# Predictors of Extraprostatic Extension in Patients with Prostate Cancer

**DOI:** 10.3390/jcm12165321

**Published:** 2023-08-16

**Authors:** See Hyung Kim, Seung Hyun Cho, Won Hwa Kim, Hye Jung Kim, Jong Min Park, Gab Chul Kim, Hun Kyu Ryeom, Yu Sung Yoon, Jung Guen Cha

**Affiliations:** 1 Department of Radiology, School of Medicine, Kyungpook National University, Kyungpook National University Hospital, Daegu 41944, Republic of Korea; 2 Department of Radiology, School of Medicine, Kyungpook National University, Kyungpook National University Chilgok Hospital, Daegu 41404, Republic of Korea

**Keywords:** prostate cancer, extraprostatic extension, magnetic resonance imaging

## Abstract

Purpose: To identify effective factors predicting extraprostatic extension (EPE) in patients with prostate cancer (PCa). Methods: This retrospective cohort study recruited 898 consecutive patients with PCa treated with robot-assisted laparoscopic radical prostatectomy. The patients were divided into EPE and non-EPE groups based on the analysis of whole-mount histopathologic sections. Histopathological analysis (ISUP biopsy grade group) and magnetic resonance imaging (MRI) (PI-RADS v2.1 scores [1–5] and the Mehralivand EPE grade [0–3]) were used to assess the prediction of EPE. We also assessed the clinical usefulness of the prediction model based on decision-curve analysis. Results: Of 800 included patients, 235 (29.3%) had EPE, and 565 patients (70.7%) did not (non-EPE). Multivariable logistic regression analysis showed that the biopsy ISUP grade, PI-RADS v2.1 score, and Mehralivand EPE grade were independent risk factors for EPE. In the regression assessment of the models, the best discrimination (area under the curve of 0.879) was obtained using the basic model (age, serum PSA, prostate volume at MRI, positive biopsy core, clinical T stage, and D’Amico risk group) and Mehralivand EPE grade 3. Decision-curve analysis showed that combining Mehralivand EPE grade 3 with the basic model resulted in superior net benefits for predicting EPE. Conclusion: Mehralivand EPE grades and PI-RADS v2.1 scores, in addition to basic clinical and demographic information, are potentially useful for predicting EPE in patients with PCa.

## 1. Introduction

Optimal counseling and treatment plans can be developed for patients diagnosed with prostate cancer (PCa) through comprehensive risk assessments [1]. Risk assessment is essential to reduce both the over- and undertreatment of patients with PCa. Patients with localized prostate cancer may experience improved outcomes with nerve-sparing robot-assisted laparoscopic radical prostatectomy (RALP), leading to reduced risk of urinary incontinence and erectile dysfunction, without an increase in the likelihood of positive surgical margins [2,3,4]. Patients with non–organ-confined PCa are at an increased risk of biochemical recurrence (BCR), and studies examining BCR after RALP have identified extraprostatic extension (EPE) as an independent predictor of BCR. Therefore, it is important to differentiate patients with organ-confined PCa from patients with non–organ-confined PCa, including patients with EPE [5].

In the clinical work-up, prostate-specific antigen (PSA) levels, digital rectal examination findings, and histopathological biopsy results are commonly applied in risk assessments. Multiparameter magnetic resonance imaging (MRI) is primarily used to outline and localize suspicious PCa lesions to perform image-guided biopsies [6]. Furthermore, aside from its capability in detecting PCa, it exhibits promising potential in preoperative staging to delineate pathological EPE and seminal vesicle involvement, with sensitivity and specificity rates of 57% and 91%, respectively [3,4,5,6].

The criteria used to assess EPE in the Prostate Imaging Reporting and Data System (PI-RADS) version 2.1 guidelines include asymmetry or the invasion of neurovascular bundles, a bulging prostatic contour, an irregular or spiculated margin, obliteration of the rectoprostatic angle, a tumor–capsule interface of greater than 1.0 cm, and breach of the capsule with evidence of direct tumor extension or bladder wall invasion [4,5,6].

Traditionally, an EPE is measured on a Likert score of 1 to 5, based on the overall subjective assessment of a combination of MRI criteria by an experienced radiologist. Recently, Mehralivand et al. [7] reported an EPE grading system based on a set of potentially less observer-dependent criteria. Against histopathologic findings as a standard of reference, the system achieved 75% sensitivity and 68% specificity for an EPE grade of 1 or greater in a retrospective study. Recent studies showed that incorporating MRI findings with clinical nomograms improved the assessment of pathologic EPE. Moreover, it is quite common to incorporate the information gained from MRI when discussing the optimal surgical and therapeutic strategies at multidisciplinary team meetings [8,9,10].

Therefore, the purpose of the present study was to investigate the effective predictors in the detection of EPE and determine how the Mehraviland EPE grading system performed compared to and in combination with other preoperative clinical variables and radiologic interpretations.

## 2. Materials and Methods

This study was approved by the Institutional Review Board (IRB) of our institution (Kyungpook National University Hospital IRB NO. 2023-06-018). The IRB waived the informed consent due to the retrospective study design. All study protocols were in accordance with the principles of the Declaration of Helsinki.

### 2.1. Patients Population

We performed a retrospective cohort study at Kyungpook National University Hospital, which is a tertiary referral hospital in Daegu, Korea. All data were extracted from each patient’s medical records. We recruited 898 consecutive PCa patients treated with RALP who underwent preoperative MRI from 1 January 2019 to 31 December 2022. Exclusion criteria encompassed individuals with a history of previous local or neoadjuvant systemic therapy for PCa or benign prostatic hyperplasia. Sixty-three patients underwent bi-parametric MRI without a dynamic contrast enhancement sequence, and thirty-five patients did not undergo multiparametric MRI due to contraindications.

### 2.2. MRI Assessment

All patients were examined using a 3.0-T MR imager according to the standardized PI-RADS v2.1 protocol. The sequences included T2-weighted images in the axial, sagittal, and coronal planes; axial diffusion-weighted images; and axial dynamic contrast-enhanced images. All prostate MRI examinations were interpreted independently and in random order during single contiguous sessions by two experienced radiologists, having 20 and 15 years of experience in prostate MRI, respectively, with both radiologists conducting approximately 500 cases per year. Importantly, the radiologists were blinded to the histopathologic examination results of the surgical specimens. All prostate MRI examinations were assessed according to PI-RADS v2.1 published by the European Society of Urogenital Radiology (ESUR), the American College of Radiology (ACR), and the AdMeTech Foundation.

The MRI EPE criteria of the pathologic EPE-curvilinear contact length with the capsule (positive when this distance > 1.5 cm), asymmetry of the neurovascular bundle (unequal appearance of the neurovascular bundles in the presence of ipsilateral PCa), capsular irregularity (unequal appearance of the neurovascular bundles in the presence of ipsilateral PCa), and capsular bulging (smooth convex extension of the margin of the prostate into the extraprostatic space, in continuity with an intraprostatic tumor) were assessed in this study.

The radiologists conducted their assessments independently without knowledge of each other’s findings. They measured tumor curvilinear contact length and tumor size on axial T2-weighted images. Additionally, both radiologists documented the PI-RADS version v2.1 scores for the predominant lesions. Subsequently, the Likert scores for EPE criteria and curvilinear contact length, obtained individually by the two blinded radiologists, were converted to EPE grades ranging from 0 to 3, as proposed by Mehralivand [10]. The MRI findings were classified according to the EPE grading system as follows: grade 0, no suspicion of pathologic EPE; grade 1, either curvilinear contact length > 1.5 cm or capsular irregularity and bulging; grade 2, both curvilinear contact length > 1.5 cm and capsular irregularity and bulging; and grade 3, frank breach of the prostate capsule or the invasion of an adjacent structure.

Neurovascular asymmetry, a potential indicator of EPE, is infrequently observed in multiparametric MRI. However, our system did not incorporate neurovascular asymmetry as a feature. Another characteristic associated with pathological EPE is the obliteration of the rectoprostatic angle. Nonetheless, this feature was excluded from the proposed standardized system for simplicity, as it only occurs in tumors located posteriorly.

### 2.3. Histopathology

Whole-mount sections were prepared from radical prostatectomy specimens via formalin fixation and cutting the specimens at 5 mm intervals. Sections with 5 mm thickness were cut from the paraffin blocks, mounted on glass slides, and stained with hematoxylin and eosin. Two uropathologists, having extensive experience of 22 and 17 years, respectively, independently delineated the presence of tumor involvement in the whole-mount section drawings. For every specimen, the uropathologists identified the presence of a pathologic index tumor and determined the tumor volume using routine pathologic measurements. Additionally, the uropathologists evaluated the number of sections with EPE in each specimen, documenting the location, maximum radial distance, and circumferential. Instances of EPE within a given specimen were further classified as either focal or non-focal EPE, following established criteria [11]. Extensive EPE was defined as an EPE with a radial extension of 1.1 mm or greater, based on the criteria proposed by Danneman et al. [5]. The findings from the histopathological analysis of the preoperative biopsies were meticulously recorded in the study database. Gleason grades and scores were subsequently grouped into five grade categories according to the 2014 International Society of Urological Pathology (ISUP) consensus conference guidelines [12].

### 2.4. Statistical Analysis

The data were summarized using descriptive statistics, presenting the median and interquartile ranges (IQRs) for continuous variables. Weighted k values were used to assess the agreement with MRI criteria between radiologists.

Primarily, a statistical comparison was made between the non-EPE and extensive EPE groups to screen for potential predictive predictors using the Mann–Whitney U-test. In the subsequent analyses, histopathologic measures (biopsy ISUP grade group) and MRI measures (PI-RADS v2.1 scores and Mehralivand EPE grades) were considered promising markers and categorized into quartiles. Subsequently, multivariable logistic regression analysis was conducted to determine the independence of these markers as predictors. Furthermore, three multivariable logistic models were employed to evaluate the potential of incorporating these predictors to enhance the accuracy of diagnosing EPE, compared to utilizing only the baseline clinical variables (i.e., age, PSA, prostate volume, the percentage of positive biopsy cores, and clinical stage).

The initial model was constructed utilizing basic clinical variables, while in the other two models, PI-RADS v2.1 scores and Mehralivand EPE grades were independently incorporated. These variables were chosen as basic clinical factors because of their ease of acquisition without requiring interpretation by a radiologist. The prognostic efficacy of each model was assessed through measures of discrimination and calibration, employing the area under the receiver-operating characteristic curve (AUC), the Hosmer–Lemeshow goodness-of-fit test, and calibration plots. To validate the predictive models, the bootstrap method was employed. For assessing the net benefit of the models, decision-curve analysis was applied. This analysis involves comparing the net benefit of a model against the reference net benefit or another model. The net benefit is determined based on the difference between the number of true-positive and false-positive results, weighted by the odds of the chosen threshold probability of risk. The reference net benefit was calculated assuming that all patients underwent EPE after RALP, while no patient experiencing EPE was assigned a zero net benefit. By comparing the net benefits at various probability thresholds, the model with the greater net benefit was deemed preferred. Data analysis, including the decision-curve analysis program provided by Vickers [13], was performed using GraphPad Prism^®^ 6.0 (GraphPad Software Inc., La Jolla, CA, USA), which is a graphical user interface for SPSS 22.0 (IBM Corp., Armonk, NY, USA) [14]. A *p*-value of <0.05 was considered statistically significant.

## 3. Results

Of the 800 patients recruited according to the inclusion criteria, 235 (29.3%) were found to have EPE. Among these, 107 patients were classified as having focal EPE, and 128 patients as having non-focal EPE. Specifically, 90 patients (38.2%) exhibited extensive EPE, characterized by a radial distance of 1.0 mm or greater. The median size of the histologic index tumor was 2.2 cc (IQR = 0.9–4.4 cc). Positive surgical margins were observed in 152 out of 800 patients (15%). Among these, 114 patients belonged to category pT3 (114/221; 51.5%), and 38 patients were in category pT2 (38/576; 6.5%). Forty-two patients had serum PSA levels of 0.02 ng/mL or greater after radical prostatectomy.

The agreement between the two radiologists was substantial, with a weighted k of 0.76 for PI-RADS version v 2.1 scores and 0.71 for Mehraviland EPE grades.

The clinical and surgical characteristics of the patients are shown in Table 1. Serum PSA levels were significantly higher in patients with EPE than in patients without EPE. Likewise, there were incremental increases in the detection rate of EPE with a high grade in the clinical T stage, the surgical ISUP grade group, and the D’Amico Risk group. We hypothesized that the biopsy ISUP grade, PI-RADS v2.1 score, and Mehralivand EPE grade could serve as predictors of an EPE in PCa patients. This section may be divided into subheadings. It should provide a concise and precise description of the experimental results, their interpretation, as well as the experimental conclusions that can be drawn.

For the purpose of identifying the optimal thresholds, we conducted calculations for observed EPE occurrences and unadjusted odds ratios related to EPE within each categorical group (Table 2). In contrast to the remaining quartiles, notably elevated odds ratios were identified within the uppermost quartile of the biopsy ISUP grade group, PI-RADS v2.1 scores, and Mehralivand EPE grades. Therefore, we used biopsy ISUP grade group 4–5, a PI-RADS v2.1 score of 5, and a Mehralivand EPE grade of 3 as optimal thresholds. The odds ratios, both unadjusted and adjusted using these thresholds, were re-evaluated. The unadjusted odds ratio for EPE was 3.12 (95% confidence interval (CI): 1.61–10.5) for biopsy ISUP grade group 4–5, 5.34 (95% CI: 1.59–16.9) for a PI-RADS v2.1 score of 5, and 11.6 (95% CI: 3.46–40.2) for a Mehralivand EPE grade of 3. After adjusting for other variables, the odds ratio for EPE was 2.23 (95% CI: 0.84–6.62) for biopsy ISUP grade group 4–5, 4.89 (95% CI: 1.12–19.7) for a PI-RADS v2.1 score of 5, and 8.23 (95% CI: 2.43–30.5) for a Mehralivand EPE grade of 3. Notably, the biopsy ISUP grade group 4–5 did not demonstrate statistical significance in the presence of other variables. Table 3 displays the adjusted and unadjusted odds ratios for EPE according to each patient factor.

Table 4 provides an overview of the predictive accuracies for the three evaluated models. The basic clinical model achieved an AUC of 0.607 (95% CI: 0.518–0.702). The addition of a PI-RADS v2.1 score of 5 to the basic model improved the AUC to 0.802 (95% CI: 0.671–0.817). However, the best discrimination was observed when Mehralivand EPE grade 3 was added to the basic model, resulting in an AUC of 0.879 (95% CI: 0.858–0.904). The enhancement of the models with the inclusion of Mehralivand EPE grade 3 was statistically significant in terms of the AUC. Both models demonstrated good calibration for EPE, as indicated by the Hosmer–Lemeshow goodness-of-fit test (*p* = 0.6171 for the model using Mehralivand EPE grade 3 and *p* = 0.3502 for the model using a PI-RADS v2.1 score of 5). The bias-corrected predictive accuracy estimated by the bootstrap method closely matched that obtained using the original data, with an AUC of 0.854 (95% CI: 0.802–0.879) for the model using Mehralivand EPE grade 3 and an AUC of 0.795 (95% CI: 0.738–0.826) for the model using a PI-RADS v2.1 score of 5.

**Figure 1:**
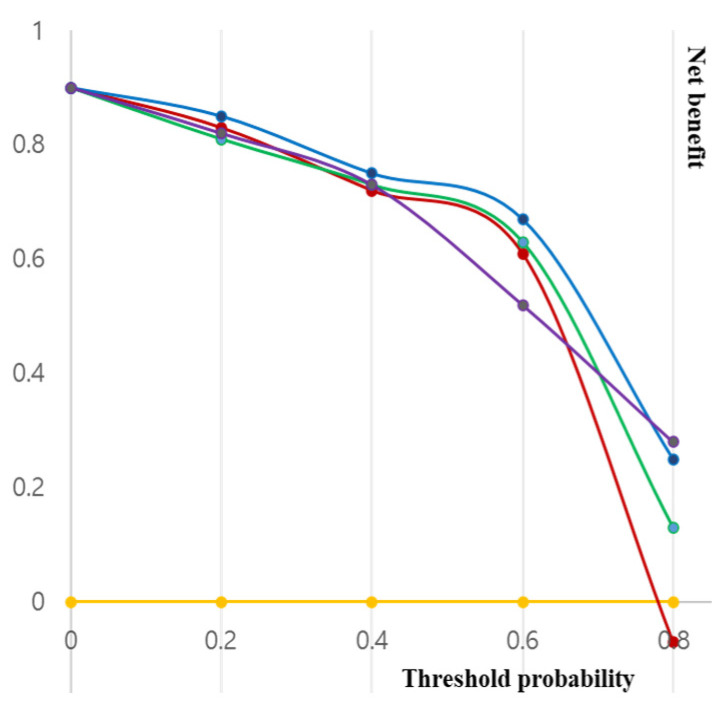
Decision curves were employed to assess the predictive capability of three analyzed models for detecting extraprostatic extension (EPE) in prostate cancer (yellow line, none; red line, all; green line, basic; purple line, model 1; and blue line, model 2). The yellow line illustrates the net benefit when assuming no patients have EPE; the red line represents the net benefit of uniformly following up with all patients, regardless of their risk, assuming universal EPE presence. The green line shows the net benefit derived from patient follow-up strategies guided by the basic model, while the blue line illustrates the net benefit resulting from following up with patients based on the basic model along with a PI-RADS v2.1 score of 5. Lastly, the purple line shows the net benefit of patient follow-up guided by the basic model coupled with a Mehralivand EPE grade of 3.

**Figure 2:**
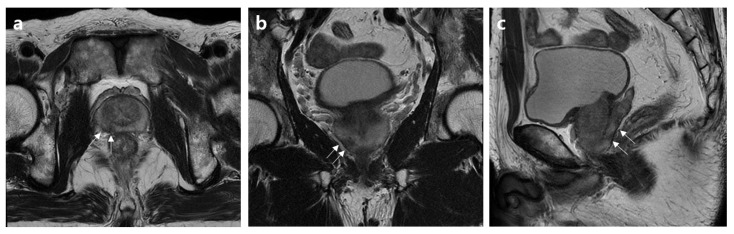
MR images of a 62-year-old patient without an extensive extraprostatic extension (EPE). (**a**) Axial, (**b**) coronal, and (**c**) sagittal T2-weighted MR images show indirect curvilinear contact length associated with a right peripheral zone mid-posterior lesion (arrows). The basic clinical findings included a serum PSA level of 16.5 ng/mL, a prostate volume of 36 mL, a positive biopsy core of 36%, clinical stage of T2c, biopsy grade 3, and intermediate D’Amico risk. The PI-RADS v2.1 scores and Mehralivand EPE grades on multiparametric MRI were 5 and 1, respectively, according to both radiologists.

**Figure 3:**
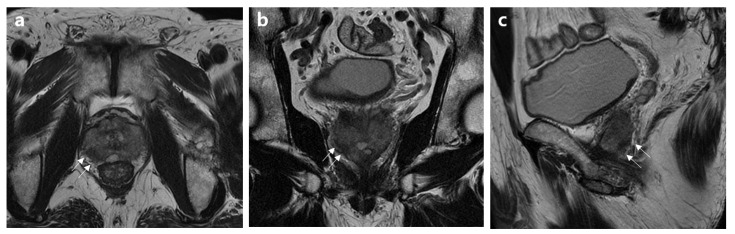
MR images of a 70-year-old patient without an extensive extraprostatic extension (EPE). (**a**) Axial, (**b**) coronal, and (**c**) sagittal T2-weighted MR images show capsular bulging or irregularity associated with a right peripheral zone mid-posterior lesion (arrows). The basic clinical findings included a serum PSA level of 15.8 ng/mL, a prostate volume of 34 mL, a positive biopsy core of 35%, clinical stage of T3a, biopsy grade 4, and high D’Amico risk. The PI-RADS v2.1 scores and Mehralivand EPE grades on multiparametric MRI were 5 and 2, respectively, according to both radiologists.

**Figure 4:**
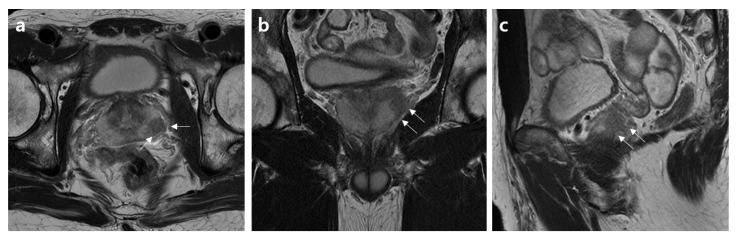
MR images of a 75-year-old patient with an extensive extraprostatic extension (EPE). (**a**) Axial, (**b**) coronal, and (**c**) sagittal T2-weighted MR images show capsular bulging or irregularity associated with a left peripheral zone mid-posterior lesion (arrows). The basic clinical findings included a serum PSA level of 15.3 ng/mL, a prostate volume of 37 mL, a positive biopsy core of 40%, clinical stage of T2b, biopsy grade 3, and intermediate D’Amico risk. PI-RADS v2.1 scores and Mehralivand EPE grades on multiparametric MRI were 5 and 2, respectively, according to both radiologists.

**Figure 5:**
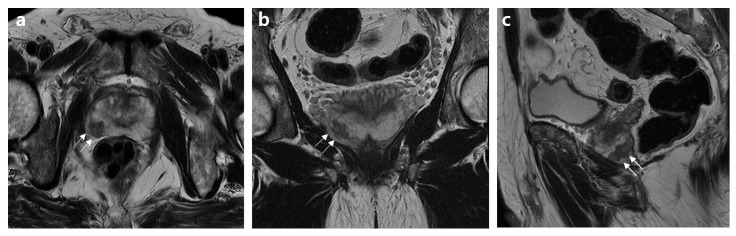
MR images of a 77-year-old patient with an extensive extraprostatic extension (EPE). (**a**) Axial, (**b**) coronal, and (**c**) sagittal T2-weighted MR images show a frank breach of the prostate capsule associated with a right peripheral zone mid-posterior lesion (arrows). The basic clinical findings included a serum PSA of 14.7 ng/mL, a prostate volume of 33 mL, a positive biopsy core of 38%, clinical stage of T3a, biopsy grade 4, and high D’Amico risk. The PI-RADS v2.1 scores and Mehralivand EPE grades on multiparametric MRI were 5 and 3, respectively, according to both radiologists.

Decision curves for EPE using the three analyzed models to assess these results in a clinical context are plotted in Figure 1. The net benefit of the basic model demonstrated either similarity to or a lower value than those of the other two models. Both the basic model and the model incorporating Mehralivand EPE grade 3 (model 1) and a PI-RADS v2.1 score of 5 exhibited greater net benefits across all probability thresholds. However, from a probability threshold of 30% onwards, the combination of the basic model with Mehralivand EPE grade 3 exhibited a higher net advantage compared to the net benefit attained through a PI-RADS v2.1 score of 5. The cumulative net advantages for the three examined models at individual probability thresholds are detailed in Table 5, and representative examples are depicted in Figure 2, Figure 3, Figure 4 and Figure 5.

## 4. Discussion

In this study, we conducted a comparison of nomograms utilizing AUC and decision-curve analysis. Decision-curve analysis showed that Mehralivand EPE grades combined with MRI were potentially useful for predicting an EPE in patients with PCa when added to clinical characteristics, such as age, PSA, prostate volume, the percentage of positive biopsy cores, and clinical stage. While the AUC metric is informative about the predictive accuracy of the model, it lacks information about clinical outcomes. Therefore, we used decision-curve analysis to offer insights into the clinical utility of a model in the absence of supplementary explicit data, such as costs or alternative assessments of clinical results.

According to a recent meta-analysis comprising 45 studies, multiparametric MRI demonstrated a pooled sensitivity of only 57% and a specificity of 91% for detecting EPE [15,16,17,18]. However, it is essential to acknowledge that the definitions of EPE observed on multiparametric MRI can vary among different readers and centers. Several imaging features have been associated with pathologic EPE, including curvilinear contact length, capsular irregularity, capsular bulging, obliteration of the rectoprostatic angle, and asymmetry of the neurovascular bundles. Notably, a visible breach of the prostate capsule or the presence of a tumor in the periprostatic fatty tissue is considered a direct and highly reliable sign indicative of a pathologic EPE [19,20,21]. However, PI-RADS version 2.1 does not yet include an explicit, highly sensitive scoring system for detecting pathologic EPEs [22,23,24]. The new Mehralivand EPE grading system can provide a template.

PI-RADS v2.1 is an MRI grading tool for detecting clinically significant prostate cancer. On the other hand, Mehralivand EPE grades are an MRI grading system specifically designed for EPE assessment. Compared to PI-RADS v2.1, Mehralivand EPE grades are considered to be more intuitive and straightforward, resulting in reduced inter-reader variability and easier implementation. While Mehralivand EPE grade 3 demonstrates clear and intuitive EPE findings, PI-RADS v2.1 score 5 includes definite MRI-visible EPE findings and also encompasses lesions corresponding to PI-RADS v2.1 score 4, with a size greater than 1.5cm. Because of this difference, Mehralivand EPE grade 3 may exhibit better performance compared to PI-RADS v2.1.

In this study, the clinical T stage was higher in patients with EPE than in patients without EPE, but the difference was not statistically significant. A previous study found that 69% of patients with clinically organ-confined PCa had organ-confined disease, and 31% had EPE in the pathological specimen [25,26,27]. This means that some patients were understaged prior to surgery. Pathologic EPE was defined as seminal vesicle involvement or the presence of any malignant cell outside the prostatic capsule. Previous studies suggested that the clinical T1 stage and pathologic T2c stage were diagnosed most frequently [23,24,25,28]. The correspondence between the clinical T stage and pathologic T category was 15% for T2a clinical stage, 10% for T2b clinical stage, and 55% for T2c clinical stage [28,29]. EPE was found in 23% of T1 clinical stage patients and 36% of T2 clinical stage. Differences in the T2a-c clinical stage subcategories were not significant [27,28,29]. Pathological EPE was not recognized clinically in >50% of patients with PCa. As with our results, there is a low agreement between clinical and pathologic T stages for localized PCa, and thus, this often leads to understaging and an insufficient prognostic prediction.

There exists a substantial association between decision-curve analysis and the risk threshold. Prior investigations showed the diagnostic performance of Mehralivand EPE grades, with an average sensitivity of 92%, specificity of 42%, and accuracy of 77% by an experienced radiologist. In this study, the diagnostic performance of EPE was found to be comparable to or slightly higher than that reported in previous studies. The Mehralivand EPE grade was thought to be the most useful predictive factor between threshold probabilities of 20% and 40%. The net benefits observed for the basic model within threshold probabilities ranging from 0% to 30% closely resembled those observed in the model, assuming EPE presence in all patients. Conversely, at a threshold probability of 35%, predictive model 2 (basic model plus Mehralivand EPE grade 3) demonstrated a net benefit 0.742 greater than that achieved by assuming all patients had EPE. This corresponds to a net rise of 74.2 true-positive outcomes per 100 patients, devoid of any accompanying elevation in the count of false-positive outcomes.

Our study had some limitations. Firstly, the sample size was relatively small, and secondly, all participants were recruited exclusively from a single research center. The limited sample size was partly attributed to the recruitment process, which focused on a single, relatively local referral hospital. Therefore, future studies should include external, larger-scaled prospective multicenter validation under the auspices of a major uroradiological society. Furthermore, MRI examinations were interpreted by readers from our hospital. As this study was conducted at a single institution, external validation is essential to evaluate the generalizability of our grading system. We do not know whether these results are generalizable to different centers because of differences in patient populations.

In conclusion, the addition of factors associated with Mehralivand EPE grades and PI-RADS v2.1 scores to basic clinical and demographic information was useful for predicting EPE in patients with PCa. Particularly noteworthy, Mehralivand EPE grades exhibited a strong correlation with EPE presence, proving notably valuable within threshold probabilities ranging from 20% to 40% according to decision-curve analysis. These findings imply that patients presenting with Mehralivand EPE grade 3 during RALP should consider extensive resection due to the potential for EPE.

Measurement of Mehralivand EPE grades may contribute to making optimal decisions regarding the management of patients with PCa diagnosed with an EPE.

## Figures and Tables

**Table 1:** Clinical and surgical characteristics of the 800 patients.

	All Patients (n = 800)	No-EPE Patients (n = 565)	EPE Patients (n = 235)	*p*-Value	Missing, n (%)
Age (years)	65.1 (61–69)	64.6 (60–67)	67.4 (59–69)	0.451	0 (0)
Serum PSA (ng/mL)	10.8 (8.2–15.8)	8.4 (7.5–17.9)	15.6 (13.5–24.8)	0.023	0 (0)
Prostate volume on MRI (mL)	42.7 (35.3–46.8)	40.3 (32.6–49.2)	42.7 (33.9–52.5)	0.562	0 (0)
MRI to surgery (day)	24.5 (11–56)	36.8 (10–53)	30.6 (12–57)	0.496	0 (0)
Positive biopsy cores (%)	33.5 (20–50)	31.3 (30–55)	37.8 (34–62)	0.097	55 (6.8)
Clinical T stage					45 (5.6)
cT1c	480	390	90	0.074	
cT2a	115	54	61	0.782	
cT2b	79	61	18	0.062	
cT2c	20	6	14	0.058	
cT3a/b	61	34	27	0.672	
ISUP grade group (biopsy)	3 (2–5)	2.1 (1.5 –2.8)	3.6 (2.8–4.2)	0.024	0 (0)
ISUP grade group (surgical)					0 (0)
1	23	18	5	0.067	
2	290	270	20	0.093	
3	273	210	63	0.105	
4	146	49	97	0.026	
5	68	18	50	0.013	
Histopathology (TMN stage)					
pT2a	42	N/A	N/A		
pT2b	23	N/A	N/A		
pT2c	511	N/A	N/A		
pT3a	171	N/A	N/A		
pT3b	50	N/A	N/A		
pT4	3	N/A	N/A		
Risk group (D’Amico)					0 (0)
Low	26	21	5	0.102	
Intermediate	496	440	56	0.089	
High	278	104	174	0.071	
PI-RADS v2.1 of the leading lesion	3 (2–5)	2 (1–3)	4 (4–5)	0.017	0 (0)
Mehralivand EPE grade	2 (1–3)	1 (0–2)	2 (2–3)	0.009	0 (0)

Note: Data are presented as median (interquartile range) unless otherwise indicated. EPE, extraprostatic extension; N/A, not applicable.

**Table 2:** Observed EPE and unadjusted odds ratios for EPE stratified by categories.

Variables	Observed EPE % (n/Total n)	Unadjusted Odds Ratios * (95% CI)
ISUP grade group (biopsy)		
1	21.7 (10/46)	Reference
2	12.4 (38/305)	3.04 (0.65 to 11.7)
3	21.3 (60/281)	2.86 (0.83 to 14.3)
4–5	75.5 (127/168)	10.3 (2.57 to 35.9)
PI-RADS v2.1 of the leading lesion		
1–2	14.1 (12/85)	Reference
3	26.4 (9/34)	1.13 (0.16 to 5.83)
4	26.6 (63/236)	7.25 (0.81 to 20.7)
5	33.9 (151/445)	13.4 (1.81 to 82.5)
Mehralivand EPE grade		
0	11.0 (11/100)	Reference
1	15.8 (24/151)	1.29 (0.33 to 5.15)
2	26.5 (98/369)	8.09 (1.55 to 8.49)
3	56.6 (102/180)	20.1 (3.67 to 72.4)

* Calculated by univariable logistic regression analysis. EPE, extraprostatic extension.

**Table 3:** Adjusted and unadjusted odds ratios for EPE according to patient factors.

Variables	Unadjusted Odds Ratios (95% CI)	Adjusted * ORs (95% CI)
ISUP grade group (biopsy) 4–5	4.12 (1.61 to 10.5)	3.23 (0.84 to 6.62)
PI-RADS v2.1 score of 5	6.34 (1.59 to 16.9)	5.89 (1.12 to 19.7)
Mehralivand EPE grade 3	12.6 (3.46 to 40.2)	9.23 (2.43 to 30.5)

* Adjusted for age, serum PSA, prostate volume at MRI, positive biopsy core, clinical T stage, and D’Amico risk group. Calculated by univariable and multivariable logistic regression analyses. EPE, extraprostatic extension.

**Table 4:** Area under the receiver-operating curves of different models, accompanied by 95% CIs and a comparison with the basic model.

Predictor	AUC (95% CI)	*p* (vs. Base)
Basic model	0.607 (0.518–0.702)	-
Model 1	0.802 (0.671–0.817)	0.023
Model 2	0.879 (0.858–0.904)	0.009

AUC, area under the receiver-operating characteristic curve. Basic model: age, serum PSA, prostate volume at MRI, positive biopsy core, clinical T stage, and D’Amico risk group. Model 1: basic model plus PI-RADS v2.1 score of 5; model 2: basic model plus Mehralivand EPE grade 3.

**Table 5:** Net benefits of the four models at each probability threshold.

Probability	All	Basic Model	Model 1	Model 2
10	0.857	0.857	0.857	0.887
15	0.754	0.754	0.762	0.845
20	0.703	0.638	0.735	0.817
25	0.686	0.626	0.723	0.799
30	0.337	0.608	0.675	0.751
35	0.226	0.279	0.523	0.742
40	−0.027	0.098	0.407	0.465

Model 1: basic model plus PI-RADS v2.1 score of 5. Model 2: basic model plus Mehralivand EPE grade 3.

## Data Availability

The dataset used and/or analyzed during the current study is available from the corresponding author upon reasonable request. The data are not publicly available because of privacy concerns.
